# Neonatal respiratory support strategies—short and long-term respiratory outcomes

**DOI:** 10.3389/fped.2023.1212074

**Published:** 2023-07-26

**Authors:** Ourania Kaltsogianni, Theodore Dassios, Anne Greenough

**Affiliations:** ^1^ Women and Children’s Health, School of Life Course Sciences, Faculty of Life Sciences and Medicine, King’s College London, London, United Kingdom; ^2^ Neonatal Intensive Care Centre, King’s College Hospital NHS Foundation Trust, London, United Kingdom

**Keywords:** mechanical ventilation, non-invasive ventilation, continuous positive airway pressure, less invasive surfactant administration, closed loop automated oxygen control, long term outcomes

## Abstract

Mechanical ventilation (MV), although life-saving, is associated with chronic respiratory morbidity in both preterm and term born infants. New ventilation modes have been developed with the aim of minimising lung injury. These include invasive and non-invasive respiratory support strategies, techniques for less invasive surfactant administration (LISA) and closed-loop automated oxygen control (CLAC) systems. Increasingly, newborn infants with signs of respiratory distress are stabilised on continuous positive airway pressure (CPAP) and receive LISA. Early CPAP when compared to mechanical ventilation reduced the incidence of BPD and respiratory morbidity at 18 to 22 months corrected age. Nasal intermittent positive pressure ventilation reduced treatment failure rates compared to CPAP, but not bronchopulmonary dysplasia (BPD). LISA compared with intubation and surfactant delivery reduced BPD, but there is no evidence from randomised trials regarding long-term respiratory and neurodevelopmental outcomes. Synchronisation of positive pressure inflations with the infant's respiratory efforts used with volume targeting should be applied for infants requiring intubation as this strategy reduces BPD. A large RCT with long term follow up data demonstrated that prophylactic high frequency oscillatory ventilation (HFOV) improved respiratory and functional outcomes at school age, but those effects were not maintained after puberty. CLAC systems appear promising, but their effect on long term clinical outcomes has not yet been explored in randomised trials. Further studies are required to determine the role of newer ventilation modes such as neurally adjusted ventilator assist (NAVA). All such respiratory support strategies should be tested in randomised controlled trials powered to assess long-term outcomes.

## 1. Introduction

Mechanical ventilation (MV) is life-saving for many preterm infants who require respiratory support in the newborn period. Unfortunately, preterm infants who require mechanical ventilation frequently develop complications (1). The most common adverse outcome is bronchopulmonary dysplasia (BPD) and importantly prematurely born infants can suffer chronic respiratory morbidity including troublesome respiratory symptoms, lung function abnormalities and exercise intolerance even in adolescence and adulthood (2). Late preterm and term born ventilated infants may also suffer complications. In a cohort of term born ventilated infants, 11% developed chronic lung disease (defined as oxygen requirement at 30 days after birth) and 9% developed neurological complications including seizures and abnormal brain imaging (3). In one study, mortality and severe neurodisability at two-year follow up were common among term born infants who required mechanical ventilation at birth affecting more than a quarter of the cohort, particularly those who were ventilated for longer than three days (4). Increasingly, studies of long-term outcomes have shown that respiratory morbidity in these groups may persist into adolescence and adulthood (5, 6). Consequently, new modes of ventilation have been developed with the aim of minimising lung injury and improving respiratory outcomes. These include a variety of invasive and non-invasive respiratory support strategies, less invasive surfactant admistration and closed-loop automated systems of oxygen saturation monitoring.

The lack of follow up studies addressing long term outcomes of neonatal ventilation strategies and the difficulties in defining outcome measures that better reflect long-term respiratory health has been previously highlighted (7). This is an important omission as it has been demonstrated that in the surfactant era there is increasing airway obstruction between 8 and 18 years in extremely preterm/llow birth weight survivors (8), thus it is important to identify interventions which might reduce/prevent such a decline. It was highlighted from a follow up study of extremely preterm infants reported in 2017, that despite substantial increases in use of less invasive ventilation after birth, there was no significant reduction in oxygen dependence at 36 weeks or improvement in lung function in childhood over time (9). We, therefore, now discuss the evidence from the most recent follow-up studies, setting this in the context of the respiratory support strategies which are currently used in neonates and how they have influenced short term outcomes. Our aims were to determine whether respiratory support techniques improve long term outcomes, highlight gaps in the literature that would help inform future research and make recommendations for clinical practice.

## 2. Non-invasive respiratory support

### 2.1. Continuous positive airway pressure (CPAP)

Continuous positive airway pressure (CPAP) delivers gas, ideally heated and humidified, with a measurable and controllable pressure transmitted through nasal prongs or a mask, connected to the infant's face. The increased positive airway pressure maintains lung expansion and prevents end-expiratory alveolar collapse. Other benefits include prevention of apnoeic episodes, improved tidal volumes and functional residual capacity (FRC) and reduced work of breathing (10). CPAP is now recommended as the optimal mode of respiratory support in infants at risk of respiratory distress syndrome (RDS) who do not require intubation for stabilisation (10). A systematic review included 3,201 preterm infants and concluded that prophylactic or very early CPAP when compared to mechanical ventilation reduced the incidence of BPD, the combined outcome of death or BPD, and mechanical ventilation (11). The use of CPAP post extubation compared to supplemental oxygen prevented respiratory failure in preterm infants, but there was no significant difference in the rate of oxygen dependency at 28 days of age (12).

CPAP should be delivered by short binasal prongs or mask with a starting pressure of 6–8 cm H_2_O (10), but there is insufficient evidence to suggest whether low or moderately high CPAP pressure levels improve morbidity and mortality in preterm infants (13). In a randomised controlled trial in preterm infants born between 26 and 32 weeks of gestation, delivery of CPAP via nasal mask when compared to nasal prongs, reduced the duration of CPAP support and the rates of moderate and severe BPD, but did not have any significant effect on mechanical ventilation requirement or the overall incidence of BPD (14). Weaning from CPAP by gradual pressure reduction increased the likelihood of successful weaning during the first attempt without increasing the total duration of CPAP support or supplementary oxygen treatment (15). A systematic review of 15 trials comparing weaning methods confirmed that, in preterm infants (<37 weeks of gestation), gradual weaning of CPAP increased the chances of success of the first weaning attempt but, by contrast with the aforementioned study, this method prolonged the weaning process. A stepdown strategy to high or low flow nasal cannula resulted in earlier weaning but it was associated with longer duration of oxygen treatment. Importantly, for none of the weaning strategies a significant effect was seen on BPD (16). Interval training, by taking the infant off CPAP for several hours each day, appears to have no clinical benefit and may increase the risk of BPD (17), and therefore should be avoided.

In a follow up study of a randomised controlled trial comparing intubation/ surfactant vs. early CPAP, treatment with early CPAP was associated with less respiratory morbidity by 18–22 months corrected age including less episodes of wheezing without a cold, respiratory illnesses diagnosed by a doctor or emergency room visits (18).

### 2.2. Nasal intermittent positive pressure ventilation (NIPPV)

Nasal intermittent positive pressure ventilation (NIPPV) is a time-cycled, pressure limited mode of non-invasive ventilation that provides two levels of pressure: a constant positive end expiratory pressure (PEEP) and a higher positive inspiratory pressure (PIP) (19). Ventilator inflations can be synchronised with the infant's breathing further improving respiratory stability (10). A 2017 Cochrane review included ten trials enrolling a total of 1,061 infants and demonstrated that early nasal intermittent positive pressure ventilation (NIPPV) in preterm infants at risk of respiratory distress within the first hours after birth reduced the risk of respiratory failure and the need for intubation compared with early CPAP without increasing the risk of complications. The use of NIPPV, however, was not associated with a lower risk of BPD (20). A more recent systematic review and network meta-analysis including 4,078 infants concluded that early NIPPV in preterm infants with RDS was associated with reduced rates of treatment failure and mechanical ventilation when compared to CPAP and h*eated, humidified, high-flow nasal cannula (HHFNC)*. Moreover, NIPPV reduced the risk of air leaks and was associated with lower rates of BPD or mortality when compared to CPAP (21).

A review of five trials demonstrated that extubation of infants to synchronised NIPPV (S-NIPPV) when compared to CPAP significantly reduced the risk of extubation failure up to one week after extubation. Non-synchronised NIPPV (NS-NIPPV) also reduced the risk of extubation failure and both modalities were associated with less air leaks. Rates of BPD did not differ between the different modalities (22). In agreement with the above, Ramaswamy, et al. reported that both S-NIPPV and NS-NIPPV reduced the risk of reintubation within the first week post extubation, but S-NIPPV was more effective than the non-synchronised mode. In that review, S-NIPPV resulted in lower rates of BPD when compared to CPAP and NS-NIPPV, whereas NS-NIPPV was the least favourable mode for that outcome (23).

### 2.3. Heated, humidified, high-flow nasal cannula (HHFNC)

Heated, humidified, high-flow nasal cannula (HHFNC) delivers heated and humidified gas at flows of between two and eight litres/ minute via nasal catheters (10). In preterm infants, HHFNC may provide positive pressure at similar levels to that used with CPAP (24). It is not possible, however, to measure the pressure delivered to the lung when using either HHFNC or CPAP and it is possible the higher treatment failure of HHFNC seen in some trials (see below) may be the result of a less effective transfer of pressure to the lung via HHFNC when shorter and smaller prongs are used compared to those used during CPAP. HHFNC has gained popularity with 87% of neonatal units reported as using it in 2015 across the United Kingdom (UK), mainly as a primary mode of post extubation respiratory support. Most practitioners preferred HHFNC due to perceived better access to the infant, less nasal trauma and quicker achievement of oral feeds, but randomised trials have not demonstrated its superiority (25). A 2016 Cochrane review concluded that HHFNC had similar rates of efficacy to CPAP either as primary or post extubation mode of respiratory support for preventing treatment failure, death and BPD, but only small numbers of extremely preterm and late preterm infants were included in the reviewed studies. Post extubation, HHFNC reduced nasal trauma and the risk of pneumothorax when compared to CPAP (24). In an international, multicentre, randomised, non-inferiority trial in 564 preterm infants born at or above 28 weeks of gestation with RDS, HHFNC as primary support resulted in a significantly higher rate of treatment failure compared to CPAP hence the trial recruitment stopped prematurely (26).

Lavizzari and co-workers demonstrated similar efficacy and safety of HHFNC to CPAP and bilevel CPAP (BiPAP) as a primary mode of ventilation in preterm infants born at greater than28 weeks of gestation with RDS (27). In agreement with this, a retrospective, two-centre observational study highlighted that the use of HHFNC for primary respiratory support in preterm infants (>28 weeks of gestation), without the use of CPAP as “rescue treatment” resulted in intubation rates within 72 h comparable to published data (14.7% and 11.1%) (28). A systematic review which included 10 trials with a total of 1,830 preterm infants found a significantly higher risk of treatment failure using HHFNC compared with CPAP (RR = 1.34, 95% CI 1.01 to 1.68), but no significant difference in intubation rates and a lower risk of nasal trauma with HHFNC. The authors concluded that HHFNC should be used as first-line option for respiratory support in centres offering CPAP/ NIPPV as a back-up (29). In a retrospective study of 134 infants, these who failed HHFNC had lower birth weight, higher inspired oxygen concentrations (FiO_2_) and maximum flow rate requirements at the time of commencing HHFNC and were more likely to be blood culture positive (30).

There is a paucity of evidence with regards to long-term outcomes of HHFNC support. A retrospective Australian study demonstrated that the introduction of HHFNC for weaning from nasal CPAP had no statistically significant effects on rates of BPD or home oxygen requirement. Infants supported with HHFNC, however, had significantly higher rates of BPD and were more likely to be discharged on home oxygen after adjusting for gestation and birth weight. The authors concluded that those findings could reflect selection bias and increased illness severity and underlined the need to evaluate the effect of HHFNC on long-term respiratory outcomes in well-designed RCTs (31).

### 2.4. Non-invasive neurally adjusted ventilator assist (NIV-NAVA)

Non-invasive neurally adjusted ventilator assist (NIV-NAVA) uses electrical signals from the diaphragm to trigger breaths and synchronise the ventilatory support with the infant's respiratory efforts (19). Studies to date have demonstrated its feasibility in preterm infants, but results are limited to short term outcomes (30). In a RCT comparing rates of treatment failure between the use of CPAP and NIV-NAVA as a primary mode of respiratory support in very low birth weight (VLBW) infants with RDS, there were no significant differences in the rates of MV, BPD and death between the two groups. The duration of MV, however, was significantly longer in the CPAP group (32). A subsequent Cochrane review identified two RCTs including a total of 23 preterm infants comparing NIV-NAVA with NIPPV. Due to the limited data, the authors could not conclude whether this mode was safe and effective in preventing respiratory failure in preterm infants (33). The available data are inconclusive with regards to the efficacy of this mode for post extubation stabilisation in preterm infants. A retrospective comparison of NIV-NAVA to NIPPV post extubation in infants born before 30 weeks of gestation did not find any significant difference in the rate of treatment failure (34). In contrast, Lee et al. demonstrated a reduction in extubation failure rate in preterm infants supported with NIV-NAVA over CPAP (35). In a more recent RCT enrolling 78 infants of less than 30 weeks of gestation, the use of NIV-NAVA post extubation was more efficient than CPAP in preventing extubation failure, but the duration of respiratory support and incidence of severe BPD were similar in the two groups (36). Further trials are required to assess the efficacy of this mode and its effect on long-term respiratory outcomes.

### 2.5. Nasal high-frequency oscillatory ventilation (NHFOV)

Non-invasive high frequency oscillatory ventilation (NHFOV) combines a continuous distending pressure through a non-invasive interface with interposed high-frequency oscillations (19) Data, however, are limited with regards to optimal settings, safety, efficacy and its impact on long-term outcomes.

In a randomised, crossover study in 30 preterm infants, transcutaneous carbon dioxide (TcO_2_) levels where significantly lower during NHFOV compared with CPAP (*p* = 0.0007) (37). Moreover, in a RCT which included 206 preterm infants extubated to either NHFOV or CPAP, NHFOV significantly reduced reintubation rates and improved carbon dioxide clearance (38). Malakian et al. randomised 124 infants born between 28 and 34 weeks of gestation with RDS to either NHFOV or CPAP as a primary mode of respiratory support and showed that the use of NHFOV did not reduce the need for mechanical ventilation during the first 72 h (*p* = 0.13), but the median duration of non-invasive ventilation was significantly shorter (37.35 vs. 49.77 h, *p* = 0.009) (39). A systematic review included eight RCTs with a total of 463 patients born at less than 34 weeks gestational age and demonstrated a lower risk of intubation along with more effective CO_2_ clearance in infants receiving NHFOV rather than CPAP. The authors concluded that the analysed trials differed in their study designs and the clinical characteristics of the study participants and, therefore, the results should be investigated in a large multicentre randomised trial (40). NHFOV might be considered before resorting to intubation and invasive ventilation because of respiratory failure on NIPPV, but this needs appropriately testing. A follow-up study aiming to explore the long-term safety of NHFOV demonstrated that preterm VLBW infants who received NHFOV after their first extubation at birth, compared with NIPPV or CPAP, did not differ significantly in the number of episodes of bronchitis, pneumonia, wheezing and re-admission rates at 24 months corrected age. In addition, pulmonary function tests and the incidence of neurodevelopmental impairment at 12 and 24 months corrected age were similar between the groups (41). The study, however, had a relatively small sample size (*n* = 139 infants) and low follow up rate (113 out of 139 infants at 12 months and 110 out of 139 infants at 24 months corrected age) and therefore further evidence is required.

## 3. Less invasive surfactant administration (LISA)

During less invasive surfactant administration (LISA), surfactant is gradually instilled in small aliquots in the trachea with the use of a thin catheter whilst the infant maintains spontaneous breathing on non-invasive respiratory support. The European consensus guidelines for the management of RDS recommend that LISA is the preferred method of surfactant administration for spontaneously breathing infants on CPAP (10). A systematic review of 14 studies concluded that LISA compared with surfactant administration through an endotracheal tube significantly reduced the risk of death or BPD at 36 weeks postmenstrual age, the need for reintubation within 72 h and the incidences of severe intraventricular haemorrhage (IVH), death during first hospitalisation and BPD amongst survivors, without increasing adverse effects (42). It should be noted, however, that appropriately designed head to head studies between LISA and INSURE have not been performed so far. Follow-up data, however, on infants who have received LISA treatment are sparse as this is a relatively new method of surfactant administration. Two-year follow up data from the first multicentre RCT on LISA [avoid mechanical ventilation study (AMV); LISA vs. endotracheal intubation intratracheal bolus surfactant] demonstrated no significant differences in growth parameters and neurodevelopmental outcomes between the LISA and control groups. There was a trend in the LISA group towards reduced episodes of bronchitis (*p* = 0.06) and the authors speculated that this may be a surrogate for improved lung function (43). Small retrospective follow up studies on LISA included 53 preterm infants less than 29 weeks of gestation and observed trends for favourable pulmonary and neurocognitive outcomes at the corrected age of three years (44). A longitudinal study on the long-term outcomes of 60 preterm infants less than 32 weeks of gestation that received surfactant via LISA or endotracheal intubation found no significant differences in hospital readmissions, severe respiratory impairment, domiciliary oxygen therapy and need for bronchodilator therapy at 24 months postmenstrual age (45). Data from the five-year follow-up of infants that received LISA in the German Neonatal Network cohort study (46) recorded in a review suggested better lung function (forced expiratory volume in one second) and improved neurodevelopmental outcomes in infants that received LISA compared with infants that received surfactant via the standard method (47). Those studies though were limited by their retrospective nature, small sample sizes and their non-randomised designs.

## 4. Invasive ventilation

### 4.1. Patient triggered ventilation (PTV)

#### 4.1.1. Assist control (Ac) and synchronised intermittent ventilation (SIMV)

Synchronisation of positive pressure inflations with the infant's respiratory efforts (patient triggered ventilation) may reduce the need for respiratory support and thereby reduce lung injury (48). During assist-control ventilation (ACV), inflations are triggered by every spontaneous breath that exceeds the critical trigger threshold. In synchronised intermittent mandatory ventilation (SIMV), only the pre-set number of inflations are triggered regardless of the infant's spontaneous breathing rate (30). A Cochrane review of studies comparing patient triggered to conventional mechanical ventilation demonstrated that ACV/SIMV was associated with shorter duration of MV, but no significant reduction in the rates of BPD, severe IVH, air leaks or mortality. AC compared to SIMV was associated with a trend to a shorter duration of weaning. Due to design of the trials, the authors could not conclude whether those benefits were due to the provision of synchronised ventilation (49). There have been no studies exploring whether AC/SIMV improved long-term outcomes.

### 4.2. Pressure support ventilation (PSV)

Pressure support ventilation (PSV) is very similar to ACV as every spontaneous breath is supported with positive pressure but, in addition, the end of spontaneous inspiration determines the termination of the ventilator inflation. The frequency is fully controlled by the patient, and, therefore, a back-up mode is required in case of insufficient respiratory drive (50). The flow is dependent on the set driving pressure, lung compliance and resistance and the inspiratory effort of the patient. The tidal volume delivered depends on the flow and the duration of the inspiratory phase (51). A Cochrane review comparing the effect of PSV and time-cycled synchronised ventilation identified only two small, randomised crossover trials which did not report on clinical morbidities or mortality (52). In a randomised controlled trial that enrolled 107 extremely low birth weight (ELBW) infants, SIMV with PS facilitated extubation and weaning during the first 28 days of life compared with SIMV alone, but there were no significant differences in the total duration of MV, the duration of oxygen treatment, or BPD and death or BPD at 36 weeks post-menstrual age. In the subgroup of infants with birth weights of 700–1,000 grams, SIMV plus PS reduced the duration of oxygen dependency (53). Erdemir and co-workers compared PSV with volume-guarantee vs. SIMV in the weaning phase of VLBW infants with RDS and found a trend towards a reduced prevalence of post-extubation atelectasis (*p* = 0.08) and lower peak inflation pressure with PSV (*p* < 0.001). There were no significant differences in the duration of weaning, rates of extubation failure, risk of leaks and rates of BPD (54). A potential reason for benefits of PSV is that this mode of ventilation supports all breaths and was shown to reduce the work of breathing in preterm infants during the weaning phase (55). In a randomised weaning trial comparing ACV and PSV, however, the median duration of weaning did not differ significantly between the groups (56). No trials have addressed the impact of PSV on long-term outcomes.

### 4.3. Proportional assist ventilation (PAV)

During proportional assist ventilation (PAV), the ventilatory support is proportional to the breathing effort of the infant. The modality uses volume and flow changes created by the patient to unload elastic and resistive work of breathing during inspiration and expiration respectively. The frequency, time and rate of lung inflations are controlled by the patient (50). Studies on PAV have only assessed short term outcomes. In a randomised crossover study, Schulze et al. demonstrated that in preterm infants with evolving BPD, PAV maintained gas exchange with lower mean airway pressures (MAP) and peak inspiratory pressures (PIP) compared with PTV, but episodes of oxygen desaturations had longer duration (57). In another study, 12 infants with a median gestational age of 25 (range 24–26) weeks were studied at a median of 43 (8–86) days. After an hour on PAV compared with an hour on ACV, the infants' work of breathing was reduced, respiratory muscle strength was higher and the oxygenation index (OI) was significantly lower (58). Subsequently, eight infants with evolving or established BPD were studied for four hours on PAV and then on ACV in random order. During PAV, their median inspired oxygen concentration (*p* = 0.049), mean airway pressure (*p* = 0.012) and OI (*p* = 0.012) were all lower (59). Most of the PAV studies have been of a crossover design and it requires experience by the clinician in applying PAV which may explain why PAV has not had much attraction over the last decade.

### 4.4. Neurally adjusted ventilator assist (NAVA)

Neurally adjusted ventilator assist (NAVA) uses the electrical activity of the diaphragm (Edi) to servo control the applied ventilator pressure. A specialised nasogastric tube with an electrode array at the distal end detects the Edi and is used as a signal to trigger the ventilator and determine the level of support. The pressure delivered throughout each inflation is proportional to the Edi signal. The NAVA level is then adjusted to increase or decrease the amount of pressure delivered per microvolt of Edi detected (60).

Studies comparing NAVA with conventional mechanical ventilation modes demonstrated that NAVA improved patient-ventilator interaction and comfort (61) and decreased PIP and MAP, work of breathing, oxygen requirement (FiO_2_) (62), sedation requirement (63) and episodes of apnoea (61). Moreover, a review of ten recent studies comparing NAVA or NIV-NAVA to conventional respiratory support modes concluded that the application of NAVA appears to be safe and feasible in premature infants as no adverse events were reported (64). In a randomised crossover study comparing NAVA with ACV in infants with evolving or established BPD, NAVA improved oxygenation by reducing OI, FiO_2_, PIP and MAP and compliance was higher (65). In a retrospective case control study, infants with evolving BPD on NAVA/ NIV NAVA had lower extubation failure rates (*p* = 0.002), shorter durations of invasive ventilation (*p* = 0.046), total duration of invasive and non-invasive ventilation (*p* = 0.026) and total length of hospital stay (*p* = 0.019). There were no significant differences, however, in the rates of BPD or home oxygen (66). A Cochrane review identified one RCT comparing NAVA with PTV in 60 preterm infants born at or above 28 weeks of gestation and concluded that there were no significant differences in the duration of MV, the length of neonatal unit stay and the rates of BPD, pneumothorax or IVH (67). The authors concluded that the study's sample size was too small to allow to detect any possible impact on long term respiratory outcomes (68). More recent data suggest that NAVA/ NIV and NAVA ventilation in preterm infants improves their growth trajectory at the time of discharge, probably due to the improved synchronisation and patient comfort (69). Large RCTS are required to explore the effect of this respiratory mode on long-term outcomes.

### 4.5. Volume targeted ventilation (VTV)

During volume-targeted ventilation (VTV), a standard volume set by the operator is delivered to the infant regardless of changes in the infant's lung function with an aim to reduce lung damage and stabilise the partial pressure of carbon dioxide (pCO_2_). VTV is difficult to apply if there is a large leak around the endotracheal tube and cuffed tubes are not in widespread neonatal practice. The level of tidal volume used significantly affects the work of breathing in preterm infants with acute RDS (70) or during weaning (71) and in infants with evolving or established BPD (72).

A systematic review of 22 studies comparing VTV with pressure-limited ventilation demonstrated that the use of VTV reduced the risk of death or BPD at 36 weeks of gestation, the rates of pneumothorax, the duration of mechanical ventilation, the rates of hypocarbia and the incidence of severe IVH and periventricular leukomalacia (PVL). There was, however, no significant difference in mortality and long term outcomes were not reported (73). The review was limited by the small sample size of most trials, the different tidal volume (Vt) delivery depending on the ventilators and the additional ventilation modes used, different timepoints at randomisation as well as different weaning approaches.

There are only a few studies of VTV compared to other ventilator modes reporting long-term respiratory or neurodevelopmental outcomes. Stefanescu and co-workers showed a non-significant trend towards benefit for the combined outcome of death or neurodevelopmental impairment at 18 months corrected age in extremely preterm infants managed with VTV (74). A randomised trial in VLBW infants receiving VTV vs. SIMV suggested no significant difference in the rate of neurodevelopmental impairment at 6 to 9 months corrected age (75). Follow up at two years of age from one of the RCTs included in the systematic review (73) demonstrated no significant differences in rates of hospital readmissions or frequency of respiratory illness between the two groups, but fewer children from the VTV arm required treatment with inhaled steroids or bronchodilators (76).

### 4.6. High frequency jet ventilation (HFJV)

During high frequency jet ventilation (HFJV), short jets of gas are released in the inspiratory circuit through a pneumatic valve, and expiration is passive. HFJV is applied in conjunction with CV, with application of PEEP. The inspiratory to expiratory ratio can be adjusted to provide fast and low volume inspirations with long expirations that could be useful in cases of hypercapnia (77). Data regarding the efficacy of HFJV in preterm infants with lung disease are limited. In an RCT comparing rescue HFJV with CV in 144 preterm infants with severe pulmonary dysfunction, there were no significant differences in mortality, chronic lung disease at 28 days and adverse effects between the two groups. In a secondary analysis up to the time of treatment crossover that included 73 infants, HFJV was associated with lower mortality, but the data were limited by the small number of the infants included in the study (78). Term infants with persistent pulmonary hypertension rescued with HFJV demonstrated improved oxygenation, but no differences in the duration of ventilation, oxygen treatment or hospitalisation compared with infants rescued with CV (79). In a retrospective case control study, the use of HFJV as a rescue treatment was not associated with a reduction in the composite outcome of death or discharge on home oxygen, but cases treated with HFJV had more severe lung disease when compared to controls introducing bias to the results (80). The most recent Cochrane systematic reviews do not support the superiority of elective and rescue HFJV over HFOV (81) or CV (78) due to the insufficient evidence that is available. Appropriately powered RCTs are required that would incorporate long term respiratory and neurodevelopmental outcomes.

### 4.7. High-frequency oscillatory ventilation (HFOV)

High-frequency oscillatory ventilation (HFOV) has been considered a lung protective ventilation strategy as it avoids atelectasis whilst minimising the risk of alveolar overdistention due to the small tidal volume delivery (82). HFOV can be used as a primary or rescue mode of ventilation (83). Higher mean airway pressure is often used during HFOV, it is, therefore, important that the clinicians have appropriate expertise if air leaks are to be minimised. Primary HFOV has been extensively studied in the context of RCTs that included preterm or low birth weight infants with pulmonary dysfunction mainly due to RDS. A systematic review included nineteen studies and demonstrated a significant, but small reduction in the risk of BPD with the use of primary HFOV, but no differences in mortality. Pulmonary air leaks occurred more frequently in the HFOV group, the risk of severe ROP was significantly reduced and there were no differences in short term neurological outcomes (84). The evidence was weakened by the significant heterogeneity of the studies included in the meta-analysis, the various interventions applied, different types of ventilators used and characteristics of the study population. Importantly, a meta-analysis of individual patients’ data from 3,229 participants did not show any advantage of HFOV over conventional ventilation in the prevention of BPD at 36 postmenstrual age and there were no significant associations with mortality or severe brain damage (85).

Better pulmonary function tests were found at one year of age among VLBW with BPD who received early HFOV at birth (86) whereas follow up data from the Provo trial at a mean age of six years showed no significant differences in the frequency of hospitalisation, pulmonary illness, asthma or disabilities (87). Follow up data from the UK Oscillation Study (UKOS), which has been the largest RCT to date comparing primary HFOV to CV in preterm infants, demonstrated similar respiratory outcomes between the groups at one year (88) and two years of age (89). The HFOV group, however, had superior small airway function when participants were assessed at 11 to 14 years (z-score for the forced expiratory flow at 75% of the expired vital capacity (FEF_75_): −0.97 with HFOV vs. −1.19 with CV; adjusted difference: 0.23 [95% confidence interval: 0.02–0.45]) and significantly higher teacher ratings for school performance (90). Those results were not subsequently confirmed by data gathered at 16–19 years of age when measures of pulmonary function were found to be similar between the groups. Participants, however, from the HFOV group were more likely to be diagnosed with asthma and to require inhalers for asthma treatment (91). A more recent prospective observational study showed that the implementation of a new ventilation care bundle with HFOV as early rescue therapy using low tidal volumes and higher frequencies increased survival free of respiratory treatment and reduced respiratory hospital admissions at two years of postmenstrual age (92).

HFOV with volume guarantee (HFOV-VG) is a promising new ventilatory mode for the treatment of respiratory failure in newborns. The clinician can set a target Vt and the ventilator adjusts the oscillation amplitude accordingly. According to a recent national UK survey, fifty-four per cent of NICUs used HFOV-VG (93) which has been shown to reduce fluctuations in tidal volumes and achieves better control of partial arterial pressures of carbon dioxide (pCO_2_) levels (94, 95). Although HFOV-VG is becoming increasingly popular among neonatal practitioners, optimal starting values have not been identified with regards to improving important clinical outcomes (96, 97).

### 4.8. Closed-loop automatic oxygen control (CLAC)

Closed-loop automated oxygen control (CLAC) systems monitor oxygen saturation (SpO_2_) values in real-time to calculate and make an adjustment to the FiO_2_ patient delivery without any human intervention (98). A literature review included 18 studies (99) and highlighted that CLAC was consistently associated with an increased percentage of time spent within the target oxygen saturation range with fewer manual adjustments to the FiO_2_ and was effective in infants on non-invasive respiratory support or mechanically ventilated at a range of postnatal ages. Results appear to be consistent for all the control algorithms (99) and across different SpO_2_ target ranges (100, 101). In addition, previous studies demonstrated that CLAC could facilitate earlier weaning of the inspired oxygen concentration when compared to manual control (102, 103). Two reviews emphasised that studies on CLAC had not reported whether the clinical outcomes of preterm infants were improved (98, 99). Most studies on CLAC included very preterm or low birth weight infants, but we have reported that in late preterm and term born infants, CLAC shared the same benefits as in the preterm population (104, 105).

More recently, a retrospective study compared two large cohorts of preterm infants admitted to a NICU before and after the implementation of CLAC as a standard of care. Mortality, morbidity and length of neonatal unit stay were not significantly different between the groups. Implementation of automated oxygen control as a standard of care was associated with shorter duration of MV, but longer duration of non-invasive respiratory support and more supplemental oxygen days (106). Neurodevelopmental outcomes at two years of age were similar between the two cohorts, but parent-reported hospital readmissions until the time of follow up were less frequent after the implementation of automated oxygen control (107). Those studies were limited by their retrospective design. Moreover, changes to standards of clinical care between the two study periods could have influenced the results. Randomised controlled trials are currently being undertaken and will provide more evidence on the effect of CLAC on clinical outcomes.

## 5. Conclusions

It is now common practice to stabilise newborn infants at risk of RDS who do not require intubation and MV on CPAP. Increasingly, less invasive surfactant administration (LISA) is used and endotracheal intubation and mechanical ventilation are avoided. Early CPAP when compared to mechanical ventilation reduced the incidence of BPD and was associated with reduced respiratory morbidity at 18 to 22 months corrected age. Delivery of CPAP via nasal mask vs. prongs may be of greater benefit and gradual vs. abrupt pressure wean seems to increase the chances of success of the first weaning attempt. NIPPV rather than CPAP or HHFNC reduced the rates of treatment failure and the need for mechanical ventilation, mortality and BPD. After extubation, NIPPV rather than CPAP reduced the risk of extubation failure, whereas HHFNC and CPAP seem to have similar efficacy. Further studies are required to assess the efficacy of newer non-invasive ventilation techniques such as NIV-NAVA and nHFOV. Furthermore, there is only limited follow-up data that have reported nHFOV as post-extubation respiratory support in preterm infants with regard to reduction in the rates of long-term respiratory morbidities and neurodevelopmental impairment compared with NIPPV and CPAP. Less invasive surfactant administration reduced the risk of death or BPD and severe IVH compared with MV, but the limited follow up studies did not demonstrate any significant improvements in long-term respiratory and neurological outcomes and growth parameters.

Synchronisation of positive pressure breaths with the infant's respiratory effort is associated with shorter duration of MV. Randomised trials of VTV have demonstrated it reduced the risk of BPD, but there were no significant differences in long-term respiratory morbidity and neurodevelopment outcomes apart from a reduction in the rates of treatment with inhaled steroids and bronchodilators at two years of age; this needs further exploration. A Cochrane review demonstrated a small reduction in BPD with primary HFOV, but the evidence was weak to support this finding. Follow up data from the UKOS trial showed that early HFOV was associated with superior lung function at 11 to 14 years, but these results were not maintained after puberty. Whether HFOV is associated with superior lung function in young adults needs testing. NAVA/ NIV NAVA in preterm infants were associated with improved growth at the time of discharge but their effect on long-term respiratory outcomes should be explored in future studies. Closed-loop automated oxygen control systems may provide a solution to the low compliance with achievement of oxygen saturation targets, but their effect on clinical outcomes of the infants needs to be determined. Testing new strategies of neonatal ventilation in RCTs that are appropriately powered to assess long-term outcomes is required. To reduce the cost, it is essential to incorporate routinely collected data. This requires excellent communication and co-operation between researchers and hospital and community practitioners. Outcomes at 18 to 24 months have been used to determine long term outcomes. The results from the UKOS study, however, demonstrated that while there were no significant effects at two years, there were significant differences at 11 to 14 years (90). Building in follow up to such an age *a priori* would likely be prohibitively expensive, unless as above routine data sources could be incorporated. Whereas it is not possible to blind clinicians during the “acute” study to the intervention and their expertise may influence the results, it is possible to “blind” those undertaking the follow up (90, 91), hence, further emphasising the importance of long term follow up to evaluate respiratory support strategies.
